# Rationale and design of the PRevention of cArdiac Dysfunction during Adjuvant breast cancer therapy (PRADA II) trial: a randomized, placebo-controlled, multicenter trial

**DOI:** 10.1186/s40959-021-00115-w

**Published:** 2021-09-27

**Authors:** A Mecinaj, G Gulati, SL Heck, E Holte, MW Fagerland, AI Larsen, E.S Blix, J Geisler, T Wethal, T Omland

**Affiliations:** 1 Department of Cardiology, Division of Medicine, Akershus University Hospital, Sykehusveien 25, 1478 Lørenskog, Norway; 2 Institute of Clinical Medicine, University of Oslo, Oslo, Norway; 3 Division of Research and Innovation, Akershus University Hospital, Lørenskog, Norway; 4 Department of Diagnostic Imaging, Akershus University Hospital, Lørenskog, Norway; 5 Clinic of Cardiology, St. Olavs Hospital, Trondheim, Norway; 6 Department of Circulation and Medical Imaging, Norwegian University of Science and Technology NTNU, Trondheim, Norway; 7 Oslo Centre for Biostatistics and Epidemiology, Research Support Services, Oslo University Hospital, Oslo, Norway; 8 Department of Cardiology, Stavanger University Hospital, Stavanger, Norway; 9 Department of Clinical Science, University of Bergen, Bergen, Norway; 10 Department of Oncology, University Hospital of North Norway, Tromsø, Norway; 11 Immunology Research Group, Institute of Medical Biology, UiT The Arctic University of Norway, Tromsø, Norway; 12 Department of Oncology, Division of Medicine, Akershus University Hospital, Lørenskog, Norway; 13 Department of Stroke, Clinic of Medicine, St. Olavs Hospital, Trondheim, Norway; 14 Department of Neuromedicine and Movement Science, NTNU, Trondheim, Norway

**Keywords:** Cardio-oncology, Cardiotoxicity, Heart failure, Breast cancer, Sacubitril/valsartan

## Abstract

**Background:**

Recent advances in the treatment algorithms of early breast cancer have markedly improved overall survival. However, anthracycline- and trastuzumab-associated cardiotoxicity may lead to dose-reduction or halt in potentially life-saving adjuvant cancer therapy. Early initiated neurohormonal blockade may prevent or attenuate the cardiotoxicity-induced reduction in cardiac function, but prior studies have been inconclusive. The angiotensin receptor-neprilysin inhibitor sacubitril/valsartan has been shown to be superior to traditional treatment in heart failure with reduced ejection fraction, but its cardioprotective effects in the cardio-oncology setting remains to be tested.

**Objective:**

To assess if sacubitril/valsartan given concomitantly with early breast cancer treatment regimens including anthracyclines, with or without trastuzumab, may prevent cardiac dysfunction.

**Methods:**

PRADA II is a randomized, placebo-controlled, double blind, multi-center, investigator-initiated clinical trial. Breast cancer patients from four university hospitals in Norway, scheduled to receive (neo-)adjuvant chemotherapy with epirubicin independently of additional trastuzumab/pertuzumab treatment, will be randomized 1:1 to sacubitril/valsartan or placebo. The target dose is 97/103 mg b.i.d. The patients will be examined with cardiovascular magnetic resonance (CMR), echocardiography, circulating cardiovascular biomarkers and functional testing at baseline, at end of anthracycline treatment and following 18 months after enrolment. The primary outcome measure of the PRADA II trial is the change in left ventricular ejection fraction (LVEF) by CMR from baseline to 18 months. Secondary outcomes include change in LV function by global longitudinal strain by CMR and echocardiography and change in circulating cardiac troponin concentrations.

**Results:**

The study is ongoing. Results will be published when the study is completed.

**Conclusion:**

PRADA II is the first randomized, placebo-controlled study of sacubitril/valsartan in a cardioprotective setting during (neo-)adjuvant breast cancer therapy. It may provide new insight in prevention of cardiotoxicity in patients receiving adjuvant or neo-adjuvant therapy containing anthracyclines. Furthermore, it may enable identification of patients at higher risk of developing cardiotoxicity and identification of those most likely to respond to cardioprotective therapy.

**Trial registration:**

The trial is registered in the ClinicalTrials.gov registry (identifier NCT03760588). Registered 30 November 2018.

## Background

As a consequence of advances in anti-cancer treatment, the burden of acute and chronic side effects may increase. A major side effect of currently established (neo-)adjuvant breast cancer therapy is cardiotoxicity, which can lead to dose-reduction or halt in potentially life-saving cancer therapy. Breast cancer patients may be at particularly high risk as they may be exposed to several cardiotoxic treatments comprising anthracyclines, trastuzumab/pertuzumab and sometimes radiotherapy in sequence [1].

Several therapeutic strategies for prevention of cardiotoxicity have been explored both in animal models and clinical trials. In particular, studies targeting neurohormonal systems known to be associated with the progression of ventricular dysfunction and heart failure, including the renin–angiotensin–aldosterone system and the sympathetic nervous system, have been conducted. In angiotensin II type I receptor knock-out mice doxorubicin does not induce cardiotoxic injury [2]. Clinical trials in humans have been conducted in order to test the hypotheses that early or preventive use of angiotensin converting enzyme inhibitors or angiotensin receptor blockers, as well as beta-adrenergic blockers, may reduce the cardiotoxic effects of anthracyclines [3–12] and trastuzumab [13–15]. Although early studies in patients receiving high-dose anthracyclines suggested a beneficial effect of intervention with angiotensin converting enzyme inhibitors, more recent studies in patients receiving lower, contemporary anthracycline doses have shown more modest effects of angiotensin blockade and no effect of beta-blockade on preventing a reduction in left ventricular systolic function [11, 12]. Furthermore, clinical trials in breast cancer patients receiving trastuzumab/pertuzumab also have yielded mixed results [13–15]. In the trastuzumab trials, preventive therapy with angiotensin antagonists or beta blockers failed to provide any benefit in the primary efficacy analyses, whereas a beneficial effect was observed for some of the secondary outcome measures [14], and in subgroup analyses of patients who had received anthracyclines prior to trastuzumab [13]. Interpretation of many of the early studies have been hampered by methodological limitations, including a non-blinded design. Moreover, even the most recent randomized, placebo- controlled studies have generally included a modest number of patients, a short duration of follow-up and used imaging techniques with a high variability such as two-dimensional echocardiography or radiation exposure such as radionuclide ventriculography. Accordingly, there is need for larger, adequately powered multicenter trials using sensitive imaging methods with low variability and longer follow-up.

Inhibition of the major neurohormonal systems by angiotensin converting enzyme inhibitors, angiotensin receptor blockers and beta blockers constitutes the basic principles of treatment of heart failure with reduced ejection fraction. A major recent advance in the treatment of heart failure has been the introduction of the combined angiotensin receptor and neprilysin inhibitor, sacubitril/valsartan, that proved superior to the angiotensin converting enzyme inhibitor enalapril in reducing the risks of deaths or hospitalization for heart failure [16].

By enhancing the endogenous compensatory and cardioprotective actions of the cardiac natriuretic peptide system and other biologically active peptides by neprilysin inhibition, stronger protection against cardiotoxicity may potentially be achieved than by angiotensin receptor blockade alone. For instance, anthracycline-induced cardiomyopathy in rodents was reduced through stimulation of intracellular pathways activated by natriuretic peptides [17, 18]. In a recent study, sacubitril/valsartan attenuated the decrease in left ventricular ejection fraction (LVEF) in a rodent model of progressive doxorubicin-induced cardiotoxicity [19]. Another in vitro study showed that administering sacubitril/valsartan during doxorubicin, trastuzumab and pertuzumab treatment prevents cardiotoxicity [20]. In a retrospective registry study [21] and an open, uncontrolled study [22], sacubitril/valsartan improved LVEF and New York Heart Association (NYHA) class and reduced levels of N-terminal pro B-type natriuretic peptide (NT-proBNP) in patients with cancer therapy-related cardiac dysfunction who were symptomatic despite treatment with optimal heart failure medication. However, the effect of sacubitril/valsartan to prevent or delay development of heart failure has not yet been tested in larger, placebo-controlled clinical trials, and no human data from randomized trials are currently available to assess its cardioprotective effect and ability to prevent subsequent cardiac dysfunction during and following adjuvant or neo-adjuvant therapy of early breast cancer. Accordingly, we hypothesized that concomitant administration of sacubitril/valsartan during treatment with anthracyclines, independent of simultaneous trastuzumab/pertuzumab, will prevent or attenuate the reduction in left ventricular function and myocardial injury compared to placebo. To address this, we designed the PRevention of cArdiac Dysfunction during Adjuvant breast cancer therapy (PRADA) II trial.

## Methods

### Study design and objectives

PRADA II is a prospective, multicenter, randomized, placebo-controlled, double blinded, parallel group, investigator initiated clinical trial evaluating the effect of sacubitril/valsartan on cardiotoxicity in patients with early breast cancer undergoing treatment with anthracyclines with or without trastuzumab/pertuzumab. Early breast cancer is defined as stages I-III, patients with stage IV (distant metastasis) are not eligible. The breast cancer treatment regimen will be according to the National guidelines for breast cancer treatment in Norway. When planning the study, depending on the immunohistochemistry markers, following treatment regimens were possible when included in the study: (1) Epirubicin 90 mg/m^2^ and cyclophosphamide 600 mg/ m^2^ (EC 90) × 4 given every 3 weeks with or without radiotherapy (2) EC 90 × 4 followed by 12-week paclitaxel of docetaxel with or without radiotherapy (3) EC 90 × 4 followed by 12-week paclitaxel of docetaxel and 12 months trastuzumab/pertuzumab with or without radiotherapy. The primary objective of PRADA II is to assess whether the administration of sacubitril/valsartan can prevent or attenuate a reduction in left ventricular systolic function expressed as change in LVEF measured by cardiovascular magnetic resonance (CMR) from baseline to 18 months. Secondary objectives include assessing whether administration of sacubitril/valsartan is associated with i) prevention of reduction in left ventricular systolic function measured by echocardiography or CMR, ii) reduced incidence of clinically significant reduction in left ventricular systolic function expressed as a reduction in LVEF ≥ 5% by CMR or a relative percentage reduction of GLS > 15%, iii) reduced incidence of cardiotoxicity, defined as: a) an absolute reduction in LVEF ≥ 10% to a value below 50% as measured by CMR or echocardiography or b) incidence of clinical heart failure, characterized by typical symptoms, iv) reduced cardiotoxic injury expressed as change in circulating concentrations of cardiac troponins I and T measured by high sensitivity assays and NT-proBNP from baseline to 18 months follow-up. Tertiary objectives include assessing whether administration of sacubitril/valsartan is associated with i) attenuation of myocardial edema and fibrosis assessed by T2-weighted short-tau inversion recovery (T2 STIR), T2 mapping and T1 mapping ii) reduced aortic stiffness assessed by pulse wave velocity measurement by CMR iii) improved functional capacity assessed by 6-min walk test and hand grip dynamometer iv) improved quality of life assessed by Chalder Fatigue Scale, the EQ-5D-5L and the EORTC QLQ-C30 questionnaire.

Primary and secondary objectives and corresponding endpoints are specified in Table 1.

**Table 1:** Objectives and endpoints in PRADA II

Primary	Objectives	Endpoints
	In patients with early breast cancer scheduled for anthracycline-containing anti-cancer therapy, to assess whether the administration of sacubitril/valsartan can prevent or is associated with attenuation of the reduction in left ventricular systolic function measured by CMR	Change in LVEF, as determined by CMR from randomization to end of blinded therapy (18 months)
**Secondary**		
	To assess whether the administration of sacubitril/valsartan is associated with:	
(1)	prevention of reduction in left ventricular systolic function measured by echocardiography or CMR	a. Change in LVEF, as determined by echocardiography from randomization to end of blinded therapy (18 months) b. Change in GLS, as determined by echocardiography from randomization to end of blinded therapy (18 months) c. Change in GLS, as determined by CMR from randomization to end of blinded therapy (18 months) d. Change in end-systolic volume measured by CMR
(2)	reduced incidence of a significant reduction in left ventricular systolic function measured by CMR or echocardiography	Incidence of clinically significant reduction in left ventricular systolic function expressed as a. An absolute reduction in LVEF ≥ 5% on CMR or b. A relative percentage reduction of GLS > 15%
(3)	reduced incidence of cardiotoxicity measured by CMR or echocardiography	Incidence of cardiotoxicity expressed as: a. An absolute reduction in LVEF ≥ 10% to a value below 50% as measured by CMR or echocardiography or b. clinical heart failure
(4)	reduced early, acute and late, chronic cardiotoxic injury measured by cardiac biomarkers	Cardiotoxic injury expressed as change in circulating concentrations of hs-cTnI, hs-cTnT and NT-proBNP

*CMR* cardiovascular magnetic resonance, *GLS* global longitudinal strain, *hs-cTn* high-sensitivity cardiac troponin, *LVEF* left ventricular ejection fraction, *NT-proBNP* N-terminal pro-B-type natriuretic peptide

The trial is registered in the ClinicalTrials.gov registry (identifier NCT03760588). It is funded by the National Program for Clinical Therapy Research in the Specialist Health Service, the Norwegian Cancer Society (project 198,136), South-Eastern Norway Regional Health Authority and the University of Oslo. Novartis will provide the study medication and matching placebo.

The study protocol, including the patient information and informed consent form, has been approved by Regional Ethics Committee and The Norwegian Medicines Agency. A data monitoring committee ensures the safe continuation of the study.

### Study participants and sample size calculations

PRADA II is a national multicenter study involving four Norwegian university hospitals, i.e., Akershus University Hospital (sponsor), Stavanger University Hospital, St. Olavs Hospital and the University Hospital of North Norway. Following written, informed consent, subjects meeting the inclusion/exclusion criteria (Table 2) are eligible for inclusion in the study. The total target enrollment is 214 patients.

**Table 2:** Main inclusion and exclusion criteria in PRADA II

INCLUSION CRITERIA	EXCLUSION CRITERIA
• Age ≥ 18 years	• Systolic blood pressure < 100 mmHg
• Women with histological evidence of invasive early breast cancer scheduled for adjuvant therapy with anti-cancer regimens that include anthracyclines	• Clear indication for ACEI, ARB or aldosterone antagonist therapy
• Eastern Cooperative Oncology Group performance status 0–1	• Contraindication for ACEI or ARB
	• Renal failure, ie. serum creatinine greater than 133 μmol/L (1.5 mg/dl) or estimated glomerular filtration rate < 45 ml/min/1.73 m^2^
	• Hyperkalemia, i.e. serum potassium greater than 5.0 mmol/L
	• Contraindication or inability to undergo CMR examination
	• Suspected poor drug compliance
	• Life expectancy < 12 months

*ACEI* angiotensin-converting enzyme inhibitor, *ARB* angiotensin-receptor blocker, *CMR* Cardiovascular magnetic resonance

Sample size calculations are based on between-groups differences for the primary endpoint. (Table 3).

**Table 3:** Power analysis and total sample size calculations assuming different changes in LVEF with alpha 0.05, power 0.8 and 0.9 and equal sized groups with a common standard deviation of 7.0%

Power 0.8				Power 0.9			
Change in LVEF%			N	Change in LVEF%			N
Placebo	Sacubitril/-valsartan	delta		Placebo	Sacubitril/-valsartan	delta	
2.6	0.7	1.9	430	2.6	0.7	1.9	574
2.6	1.4	1.2	1072	2.6	1.4	1.2	1434
**3.4**	**0.7**	**2.7**	**214**	3.4	0.7	2.7	286
3.4	1.4	2.0	388	3.4	1.4	2.0	518
4.2	0.7	3.5	128	4.2	0.7	3.5	172
4.2	1.4	2.8	200	4.2	1.4	2.8	266

*LVEF* left ventricular ejection fraction

A larger standard deviation in change in LVEF than observed in the first PRADA trial accounts for the loss of power associated with a higher drop-out rate. With a common standard deviation (SD) of 7% and 214 patients included, PRADA II will have 80% power to detect a difference in change in LVEF of 2.7%. Identifying subgroups of high-risk patients is of great interest. Baseline cardiac troponins, indicating chronic myocardial injury, may potentially assist in discriminating between those who will benefit from those who will not benefit from the intervention. Based on the assumption from PRADA, PRADA II is also powered (68%) for the primary endpoint in the subgroup of patients with baseline troponin I/T > sex adjusted 99 percentile value (anticipated to 35% of total sample). The nominal significance level is set to 5%.

### Randomization and intervention

Patients will be randomized in a 1:1 ratio to the interventional versus control group, i.e. sacubitril/valsartan versus placebo. The randomization procedure will be performed electronically through the eCRF (i.e. Viedoc). The randomization list will be stratified according to study site and scheduled treatment with trastuzumab/pertuzumab. Block randomization will be used, with block sizes 4, 6, and 8, in random order.

Sacubitril/valsartan (target dose 97/103 mg b.i.d.) and matching placebo will be provided orally in a 1:1 parallel fashion stratified by study site and for planned treatment with trastuzumab/pertuzumab. Dose titration will be performed as follows: sacubitril/valsartan 24/26 mg b.i.d. will be administered for 2–4 weeks and provided a systolic blood pressure > 100 mm Hg, no symptoms of hypotension or other side effects or adverse events, followed by sacubitril/valsartan 49/51 mg b.i.d. for 2-4 weeks. Provided systolic blood pressure > 100 mm Hg, no symptoms of hypotension or other side effects or adverse events, a further uptitration to sacubitril/valsartan 97/103 mg b.i.d. will be performed. The duration of blinded therapy will be 18 months and will continue until the final imaging exam has been completed.

### Study procedure and follow-up

**Fig. 1:**
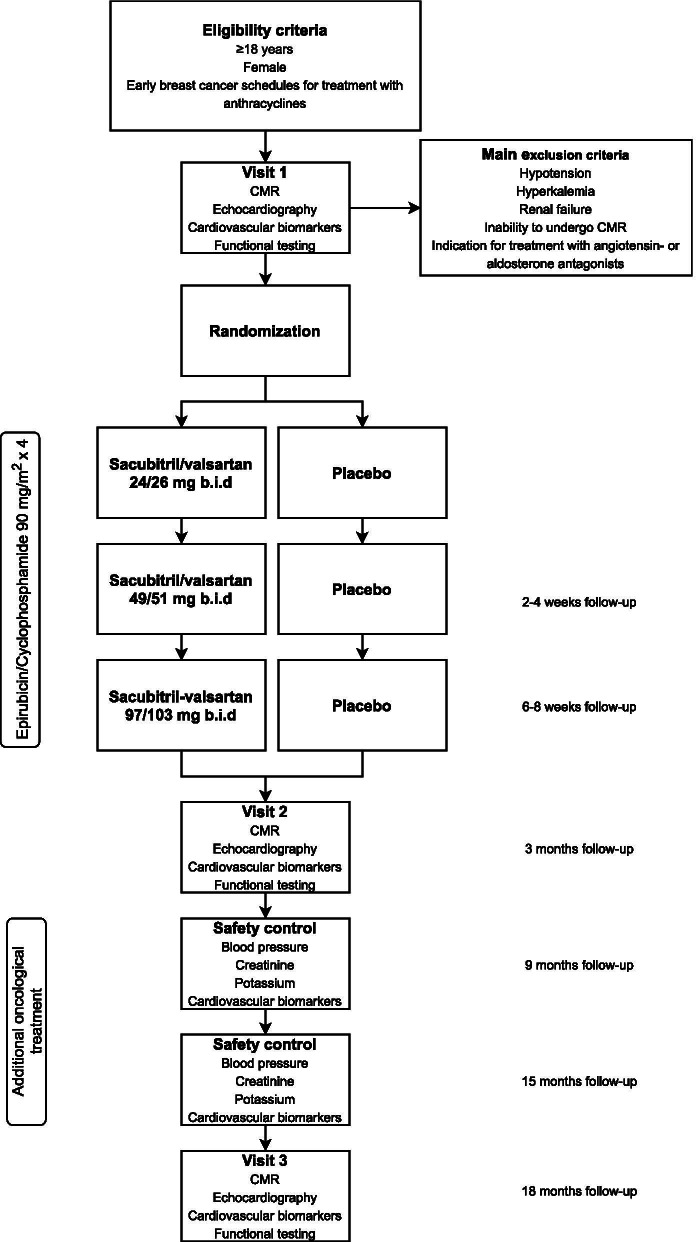
Rationale and Design of the PRevention of cArdiac Dysfunction during Adjuvant Breast Cancer Therapy (PRADA II) trial: Flow chart. CMR = Cardiovascular magnetic resonance B.i.d = twice a day

The patients will be examined with CMR, echocardiography, circulating cardiovascular biomarkers and functional testing at baseline, at end of anthracycline treatment and at 18 months (Fig. 1). Adverse events will be registered during telephone and study visits.

#### CMR

Depending on the availability at each center either a Siemens or Philips 1.5 T scanner will be used. All examinations will include breath-hold Steady-State-Free-Precession sequences (SSFP) in contiguous short-axis images covering the entire ventricles for assessment of myocardial function, volumes and mass, as well as left ventricular long axis slices in 2, 3 and 4- chamber views. Myocardial edema and fibrosis will be assessed by T2 STIR and T2 and T1 mapping, each in one mid-ventricular short axis slice. Aortic stiffness will be assessed by the pulse wave velocity by phase-contrast CMR imaging perpendicular to the ascending and descending aorta, as well as oblique-sagittal image of the aorta. CMR analyses will be performed by a limited number of expert reviewers at a dedicated CMR core laboratory at Akershus University Hospital. Images will be stored digitally for offline analysis. All CMR assessments will be performed according to the Society for Cardiovascular Magnetic Resonance recommendations [23, 24].

#### Echocardiography

All centers will be performing a transthoracic echocardiography using a Vivid E95 (GE Vingmed, Horten, Norway). Standard recordings of two-dimensional (2D) cine loops always with a minimum of three consecutive heartbeats will be recorded in standard parasternal and apical views. Additionally, pulsed and continuous wave Doppler recordings, tissue Doppler recordings, M-mode recordings and three-dimensional (3D) recordings will be made. All analyses will be performed offline on custom software (EchoPAC, GE Vingmed, Horten, Norway).

The core laboratory at St. Olavs Hospital, Trondheim University Hospital will be responsible for the echocardiography analyses. Images will be stored digitally for offline analysis. All assessments will be performed according to the guidelines or recommendations from the American Society of Echocardiography and the European Association of Cardiovascular Imaging [25, 26].

#### Biomarkers

Blood samples for biobanking will be drawn at each main visit. Cardiac troponin I and T analyses will be performed using high sensitivity assays at the clinical chemistry laboratory of participating hospitals at each study visit and at each EC 90 cycle. Changes in concentrations of NT-proBNP and cardiac troponin represent secondary study endpoints. In addition, exploratory analyses of new biomarkers will be performed. Biobank samples will initially be stored locally before batch transfer to the core biobank at Akershus University Hospital.

#### Functional testing

The 6-min walk test and hand grip dynamometer test will be performed at each main visit. The procedure is according to the American Thoracic Society Guidelines [27].

#### Quality of life

The patients will be asked to fill out the Chalder Fatigue Scale, the EQ-5D-5L and the EORTC QLQ-C30 questionnaire at each main visit.

### Statistical considerations

The main analyses are planned when all patients have concluded the study, all data have been entered, verified and validated and the database has been locked.

The primary variable (LVEF as measured by CMR) will be analyzed with a linear mixed model with treatment group, time, treatment group x time interaction, and stratification factors (trastuzumab therapy and study site) as fixed effects. A random intercept will be used. The primary variable is measured at three time points: visit 1 (baseline), visit 2, and visit 3. To allow for separate effects of time from visit 1—> visit 2 and visit 2—> visit 3, time will be modelled as piecewise linear with a knot at visit 2 in the linear mixed model. Based on the fitted model, means and 95% confidence intervals will be estimated for each treatment group for each time point, the change from visit 1 to visit 3 for each treatment group, and the between-treatment difference in change from visit 1 to visit 3 (the primary outcome). A p-value for the test of no between-treatment difference will also be reported.

The primary analysis will be performed on the intention to treat (ITT) sample. A secondary analysis will be performed on the per-protocol (PP) sample. Intra- and inter-observer agreement of LVEF measurements will be evaluated by intraclass correlation coefficient.

Secondary endpoints will be analyzed with linear mixed models (continuous variables measured at more than two time points), linear regression (continuous variables measured at two time points), logistic regression (dichotomous variables), or other suitable statistical methods.

In case of severe unbalance in important baseline values or characteristics, sensitivity analyses will be performed using the unbalanced baseline variables as an adjustment factor in the primary statistical model. The decision to perform such sensitivity analyses will be done post-hoc based on the severity of the unbalance and the assumed prognostic strength.

Details of the statistical analyses, including how to handle missing data and definitions of the analysis samples (ITT and PP) and all subgroup and sensitivity analyses, will be provided in a statistical analysis plan, to be completed before database lock.

If there are substantial missing data attributed to the discontinuation of medication or not reaching target dose (due to withdrawal of patient) then sensitivity analyses will be performed with different methods for handling missing data. The decision to include such analyses will be made based on actual data but before the database is locked and the blind is broken.

### Safety management

Patients are monitored for adverse events attributable to the study medication at every main study visit. The Common Terminology Criteria for Adverse Events version 4.0 will be used for reference. In addition, blood samples for serum creatinine and potassium will be obtained at each study visit and in conjunction with each EC cycle. Vital signs including heart rate, systolic and diastolic blood pressure and body weight will be noted. Additionally, participants will be equipped with fully automatic blood pressure monitors during Investigational Medical Product uptitration and will be asked to keep recordings at specific days.

## Results

The study is ongoing. Results will be published when the study is completed.

## Discussion

Anthracycline- and trastuzumab/pertuzumab-induced cardiotoxicity is a clinically important side effect that may result in interruption or halt in potentially lifesaving (neo-)adjuvant cancer therapies. A study published in 2015 indicated that early initiated neurohormonal blockade with angiotensin converting enzyme inhibitors and beta blockers may reverse or attenuate decline in cardiac systolic function in cancer patients receiving anthracyclines [28]. However, several small-scale randomized controlled clinical trials in early breast cancer patients have showed none or only a minor effect of angiotensin and/or beta blockers [3–10]. This may reflect that successful implementation of low-to moderate anthracycline doses and sequential anthracycline and trastuzumab therapy have reduced the risk of cancer therapy related cardiac dysfunction but may also be due to methodological issues. Larger studies with greater statistical power to detect minor to moderate changes and between-group differences in ventricular function and more efficient interventions are therefore required to answer the question whether neurohormonal blockade is efficient in preventing a cardiotoxicity-induced reduction in cardiac function. The prospective, multicenter, randomized, placebo-controlled, double blinded PRADA II clinical trial is therefore designed to address the shortcomings of prior studies by using a multicenter design, the reference method for assessing cardiac function and structure; CMR, novel echocardiography parameters and a novel and more complete neurohormonal antagonist as the intervention; sacubitril/valsartan. There are core laboratories for blinded CMR and echocardiography analysis and for biomarker analysis.

### Choice of outcome measures

The American Society of Echocardiography and the European Association of cardiovascular Imaging define cancer therapy-related cardiac dysfunction as an absolute reduction in LVEF ≥ 10% to a value  < 53% [29]. The criterion of a reduction of 10% is based on the high variability of 2D echocardiographically determined LVEF [29]. With the use of more accurate imaging methods such as CMR, the coefficient of variation is lower and in PRADA II we have based on our experience from PRADA defined a significant reduction in LVEF measured by CMR as 5% or greater. Recognizing that even smaller reductions in LVEF may have prognostic implications, the between group difference in LVEF as a continuous variable will be used as the primary outcome measure. As contemporary echocardiographic measures such as GLS and 3D LVEF may be more sensitive indices of cardiotoxicity than 2D LVEF, these will represent secondary or predefined exploratory outcome measures.

Although cardiotoxicity is commonly defined as a reduction in cardiac function measured by LVEF, this reduction may more appropriately be considered an effect of cardiotoxicity rather than cardiotoxicity per se. A more direct measure of myocardial injury is biochemical evidence of cardiomyocyte necrosis or injury by measurement of cardiac troponins by high-sensitivity assays. Cardiac troponin data may therefore represent both a more direct and a more sensitive index of cardiotoxic injury than imaging measurements of cardiac function, which may be affected by compensatory hemodynamic mechanisms. Supporting this notion, data from the PRADA [12] and CECCY [11] (Carvedilol for prevention of chemotherapy-related cardiotoxicity) trials show that intervention with beta-blockade resulted in a significant attenuation of the cardiac troponin response to anthracyclines in both studies, whereas no effect on change in LVEF was observed. Finally, with the realization that the functional capacity and subjective well-being of patients are not necessarily reflected in objective measurements of cardiac injury and function, PRADA II will include both patient-reported data on quality of life and fatigue, as well as functional testing by the 6 min walk test and grip strength testing.

### Choice of intervention

When the PARADIGM (Prospective Comparison of ARNI with ACEI to Determine Impact on Global Mortality and Morbidity in Heart Failure) trial was published in 2014 [16], sacubitril/valsartan was commonly considered the first great advance in pharmacological therapy for patients with chronic heart failure and reduced ejection fraction during the past two decades. Sacubitril/valsartan was shown to significantly reduce cardiovascular mortality and hospitalizations due to heart failure in adult patients with reduced LVEF when compared to enalapril. Subgroup analyses of patients with chronic heart failure and preserved ejection fraction in the PARAGON (Prospective Comparison of Angiotensin Receptor Neprilysin Inhibitor With Angiotensin Receptor Blocker Global Outcomes in HFpEF) trial suggest that patients with heart failure and mild reduction in LVEF may benefit form sacubitril/valsartan [30], raising the exciting possibility that this intervention may be helpful in attenuating a cardiotoxicity-associated reduction in systolic ventricular function. Currently, limited data are available concerning the effect of sacubitril/valsartan in the cardio-oncology setting, and no preventive, randomized trials have been performed in humans. In a small case-series of two patients with anthracycline-induced cardiomyopathy, treatment with sacubitril/valsartan was associated with some recovery of left ventricular function and normalization of NT-proBNP concentrations [31]. In a retrospective multicenter study of 67 patients with cancer therapy-related cardiac dysfunction from Spain, sacubitril/valsartan was associated with improvement in echocardiographic structural and functional parameters and a reduction in NT-proBNP concentrations [21]. Experimental data from animals also suggest that sacubitril/valsartan protects against anthracycline-induced cardiomyopathy in mice and that the effect is partly associated with alleviating Drp1-mediated mitochondrial dysfunction [32]. In summary, sacubitril/valsartan seems to have beneficial effects both in animal models and in established cancer therapy-related cardiac dysfunction, but randomized clinical trial data in the preventive setting are missing.

### Targeting high-risk populations in cardio-oncology

With the use of moderate to low doses of anthracyclines and routine monitoring of left ventricular function in patients using trastuzumab/pertuzumab, the incidence of cancer therapy-related cardiac dysfunction has declined substantially. The strategy to broadly administer preventive therapy has therefore been debated [33]. Ideally, pharmacological interventions should be targeted to those at highest risk and those who will benefit from the intervention. Several monitoring strategies have been proposed using early echocardiographic or biochemical markers of myocardial injury [34]. However, a limitation of these strategies is that the cardiotoxicity may already have occurred at the time of detection. Moreover, it remains an open question whether or not these strategies provide the sufficient level of prognostic accuracy to identify patients who will develop cancer therapy-related cardiac dysfunction. In PRADA II we have prospectively defined a subgroup of patients with elevated cardiac troponin concentrations at baseline and will assess whether this simple biomarker test is able to discriminate between those who will benefit from those who will not benefit from the intervention. Acknowledging that susceptibility to cardiotoxic drugs may vary, we will also assess the prognostic value of genetic, proteomic and metabolomic factors.

## Conclusion

The academic field of cardio-oncology is still comparably new and with rapid developments in clinical oncology and cardiology, the cardio-oncologic landscape is continuously shifting. In early breast cancer, novel anthracycline formulations like pegylated liposomal doxorubicin and novel anthracycline-avoiding strategies like the taxotere and cyclophosphamide chemotherapy are only some examples of recent changes that will have implications for clinical trials aiming to prevent or attenuate cardiotoxicity. These changes also imply that studies in the cardio-oncology field published many years ago, may have less relevance for current clinical practice. So far, the results of preventive clinical trials in cardio-oncology have been inconsistent and there is clearly a need for new and well-conducted trials with novel and more potent interventions to provide robust results and bring the field forward. PRADA II is designed with the aim of providing such information.

## Data Availability

Not applicable.

## Abbreviations

CMR: Cardiovascular magnetic resonance
EC 90: Epirubicin 90 mg/m^2^ and cyclophosphamide 600 mg/ m^2^
ITT: Intention to treat
LVEF: Left ventricular ejection fraction
PP: Per-protocol
